# Morphological diversity in purple nutsedge from four agro-ecological zones in Ghana

**DOI:** 10.1016/j.heliyon.2021.e07661

**Published:** 2021-07-24

**Authors:** J.W. Tachie-Menson, J.N. Buah, M.O. Adu, E. Afutu

**Affiliations:** Department of Crop Science, College of Agriculture and Natural Sciences, University of Cape Coast, Cape Coast, Ghana

**Keywords:** Noxious weeds, Purple nutsedge, Diversity, Agro-ecology, Weed management

## Abstract

Purple nutsedge (*Cyperus rotundus* L.) exhibits plasticity and are morphologically different under different conditions. Due to these differences, the weed might respond differently to weed management. Here, we examined the morphological characteristics of purple nutsedge from southern Ghana relative to those reported from other countries and further assessed differences in ecotypes from four agro-ecological zones in Ghana. A total of 46 purple nutsedge samples: 40 samples from 40 communities across the study area and three each from two research stations were used for the study. The plants were multiplied, planted into pots and laid out in Completely Randomized Design with three replications. Qualitative and quantitative assessments were carried out on both underground and aboveground morphological traits of the weed samples. The qualitative traits of the samples generally conformed to those reported in other countries and did not vary significantly between the agro-ecological zones (P > 0.05). The quantitative (growth) parameters showed significant differences among agro-ecological zones (P < 0.05) and were generally smaller than those reported in other countries, suggesting morphological adaptation of the weed in Ghana. Samples from the transitional zone were significantly smaller and this could facilitate easier management of the weed in that area. The principal component analysis gave four latent factors, which mainly pointed to photosynthetic structures and growth characteristics as the major components determining variations in the collection. Cluster analysis gave four clusters (at 0.7 similarity index), which were related neither to their geographic origin nor to the agro-ecological zones.

## 1 Introduction

Purple nutsedge (*Cyperus rotundus* L), a prominently noxious weed of global importance was rated as the world's most important weed in the late 1970s (Holm et al., 1977). Purple nutsedge exhibits prolific reproduction, grows quickly and competes very well with most crops (Horowitz, 1972; Iqbal et al., 2012). It thrives very well in most soils and stores substantial quantities of nutrients in its tubers, which also serve as vegetative reproductive structures (Akobundu and Agyarkwa, 1987; Doll, 1994). These attributes make the weed especially difficult to manage in farmers' field and other places of importance.

The weed, has for several years, been a major problem to farmers, mostly vegetable growers in Ghana. According to Tachie-Menson (2016), farmers complained of its adverse effect on the growth and yield of their crops and the difficulty in finding effective management strategies for it. Some farmers had abandoned large parcels of infested arable land due to the ineffectiveness of the management strategies they employed. Yet in some other areas, the same approaches were said to be effective in managing the weed. This raises concerns about variation in this weed species, and how it influences the efficiency of the control measures employed.

Effective weed management strategies are not one-size-fits-all for all weeds due to variations in weed morphology, growth habit and life cycle. Thus, morphological differences in weed species can render an otherwise effective management strategy ineffective. For instance, Zimdahl (2007) indicated that the effectiveness of herbicides is greatly influenced by leaf characteristics such as length and width of leaves, leaf orientation and leaf surface characteristics such as the presence of wax and hairs. In addition to inter-species differences, individual weed species also exhibit morphological differences that can cause differences in the weed's response to management practices.

Within-species diversity has been observed and repeatedly reported in various characteristics of purple nutsedge across the globe (Pena-Frontera et al., 2009; Stoller and Sweet, 1987; Wills, 1998). Diversity in purple nutsedge has, for example, been reported in tuber dormancy, development and longevity, rhizome development, the onset of flowering and response to herbicides (Stoller and Sweet, 1987). These variations have been noted to be particularly pronounced among samples from different locations (Wills, 1998). Variations in purple nutsedge have led to a description of ecotypes, such as those based on glume colour (Ranade and Bums, 1925); chemotypes, such as those from Japan and China based on sesquiterpenes in tubers (Komai and Ueki, 1981) and soil moisture (Pena-Frontera et al., 2009). Wills (1998) reported various reproductive and morphological differences in purple nutsedge collections from 13 states within the United States and 21 other locations around the world. Lye (1983) also proposed four subspecies from East Africa: subsp. *rotundus*, subsp. *merkeri*, subsp. *taylorii* and subsp. *tuberosus* based on the morphological difference observed. Similarly, Juffe (2010) suggested four subspecies of the weed: *C. rotundus rotundus*, *C. rotundus divaricatus*, *C. rotundus merkeri* and *C. rotundus retzii*. Morphological variations in a weed species are influenced by genetic differences caused by recombination during sexual reproduction, mutation and local adaptation to environmental conditions (Arteaga et al., 2015; Lascoux et al., 2016). The different biotypes or ecotypes may portray different population dynamics and relate differently to the environment. This may influence its competitive ability in the plant community and its response to management regimes.

The differences in levels of noxiousness and difficulty in the management of the purple nutsedge as indicated by Tachie-Menson (2016) could be due to the existence of different ecotypes of the weed in different agro-ecological zones in the country. In this study, we documented the morphological characteristics of purple nutsedge from southern Ghana relative to those from other locations across the globe. We assessed the morphological differences between purple nutsedge plants obtained from four agro-ecological zones in southern Ghana.

## 2 Materials and methods

### 2.1 Study area

**Figure 1:**
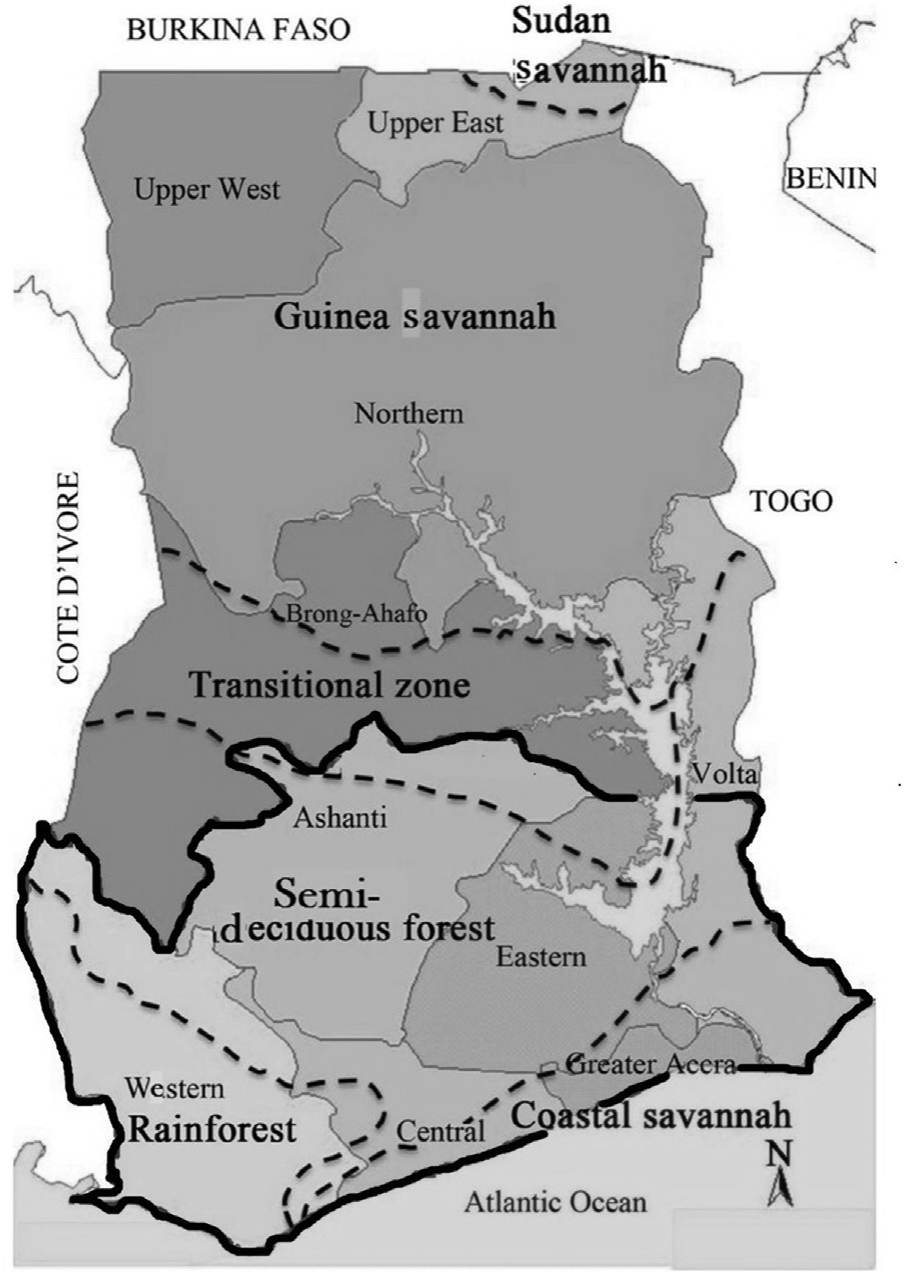
Map of Ghana showing the regions and agro-ecological zones in the study area marked by the solid black line (adapted from Aboagye et al. (2016).

The study was conducted in southern Ghana which is made up of six political regions: Western, Central, Greater Accra, Eastern, Ashanti and Volta. The varied climatic conditions in these regions separate the area into four agro-ecological zones namely, the tropical rainforest which covers some parts of the Western and Central Regions; the semi-deciduous forest zone, which covers the other parts of the Western Region, most parts of the Ashanti and Eastern Regions and the middle portions of the Volta region; and the coastal savannah which covers the other parts of the Central Region, Greater Accra and the lower parts of the Volta Region. The northern part of the Eastern and Ashanti regions fall in the transitional zones between the semi-deciduous forest and the guinea savannah zones, the predominant ecological zone in northern Ghana (Figure 1). Experiments for the characterization were conducted from January 2016 to March 2016 at the Technology Centre of the University of Cape Coast situated in the coastal savannah agro-ecological zone with annual rainfall averaging 800mm with minimum and maximum daily temperatures averaging 22.4 °C and 30.6 °C respectively (FAO, 2005).

### 2.2 Layout and experimental design

Tubers of purple nutsedge, averaging 25 in number, were collected from 3 to 5 points (depending on the abundance of the weed in the locality) in each of 46 locations. The samples for each location were then bulked, bagged and sent to the experimental site for the study. The 46 locations comprised 40 communities across the study area and three experimental plots from each of two research stations: Teaching and Research Farms of the University of Cape Coast, located in the coastal savanna agro-ecological zone and the demonstration field of the Department of Horticulture of the Kwame Nkrumah University of Science and Technology located in the semi-deciduous forest. Ten (10) tubers of the purple nutsedge from each of the locations, were first planted in 28 cm diameter plastic pots filled with loamy soil and allowed to grow and multiply for 3 months. The pots were watered as and when necessary and other weeds removed from the pots by handpicking. This was necessary to ensure a uniform start for all the tubers used in the experiment and eliminate the effect that tuber age might have on sprouting and subsequently on its morphological characteristics.

Following multiplication, 12 new mature tubers with no sprouts were selected at random for each location and four tubers were planted per pot of 28 cm diameter and 15 cm depth. The pots contained a mixture of loam, sand and decomposed poultry manure in the ratio of 4:1:1. The pot experiment which was carried out on a concrete platform in an open place, and was laid out in a Completely Randomized Design with 46 treatments and three replications.

The soil medium for the experiment was obtained from a source that had no record of the weed under study. The absence of the weed was confirmed before planting of the tubers by a careful assessment of soil samples for the presence of the tubers and later, by allowing the germination of the weed seed bank of the soil. The soil was analysed for its physical and chemical properties at the Soil Science Laboratory of the same institution, using standard protocols as follows: Total nitrogen in the soil was determined using the micro Kjeldahl method as described by Stewarte et al. (1974). Available phosphorus content in the soil was analysed following the Bray-1 acid method (Maghanga et al., 2015). The soil organic carbon (SOC) content was determined by the modified wet oxidation method (Nelson and Sommers, 1996). The soil pH was determined using the glass electrode of a Suntex SP-701 pH meter in 1:2.5 soil: water (w/v) suspension. The hydrometer method (Ashworth et al., 2001) was used to analyse the soil particle size distribution and the textural class of the soil was determined with the USDA textural triangle. Table 1 shows the chemical and physical properties of the soil.

**Table 1:** Soil properties of potted soil media.

Soil Property	Level in soil
% Nitrogen	0.09
Phosphorus (*μ*gP/g)	5.86
Potassium (cmol/kg)	0.31
% Organic carbon	0.90
pH	6.2
Bulk density (g/cm^3^)	1.29
Textural class	Sandy loam

### 2.3 Data collection

**Figure 2:**
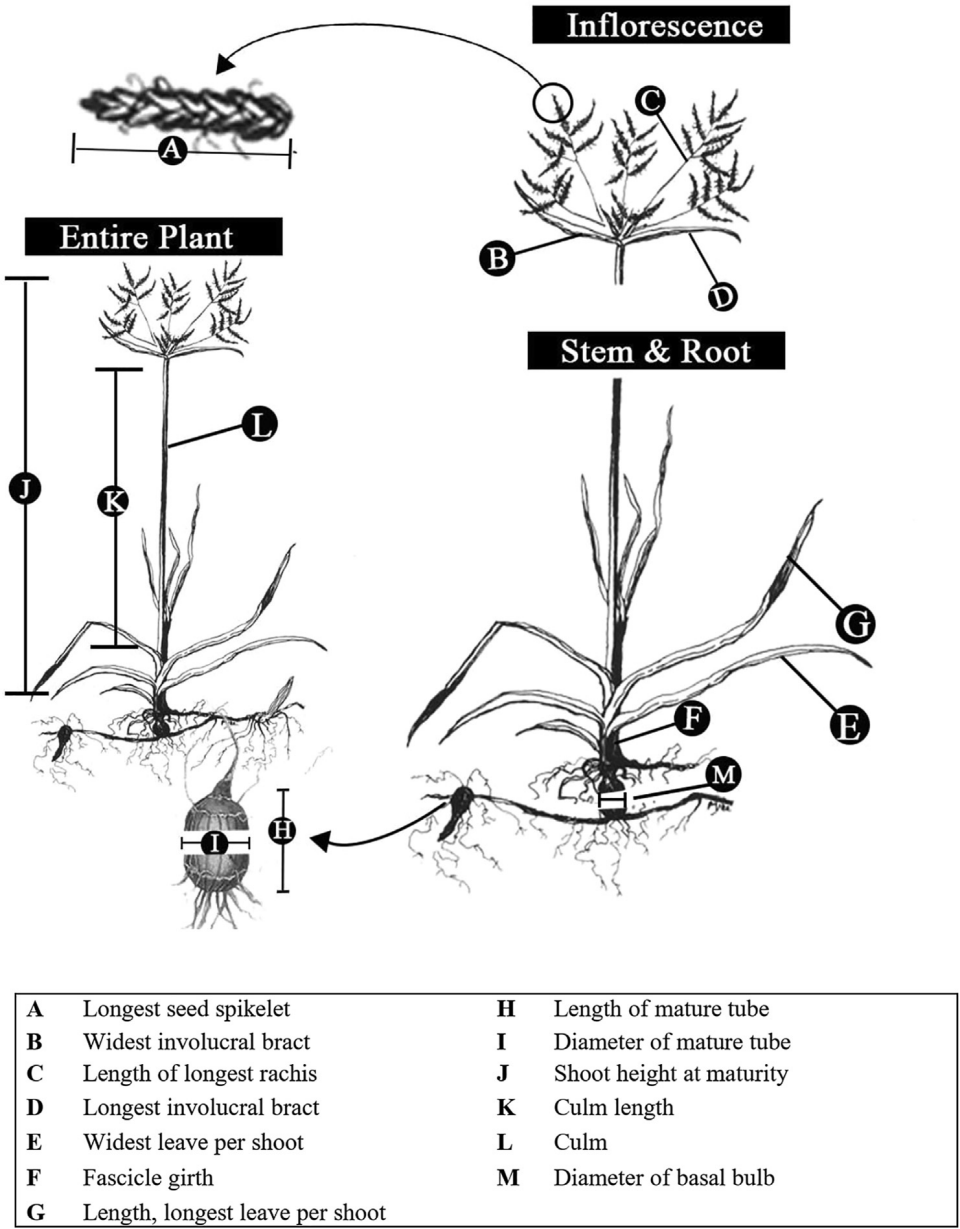
Purple nutsedge showing the quantitative characters measured (Pictures were adapted from www.flickr.com/photos/uhmuseum/).

The plants grew for three months. They were subsequently characterized based on the underground and above ground (including inflorescence) characteristics. Data were collected on five randomly selected mature tubers and shoots per pot as follows: Skin colour of mature tubers was rated on a scale of 1 (brown) to 10 (black) with the aid of a colour chart based on the RGB model. The shape of mature tuber was scored on a nominal scale of an oval (1), oblong (2), ovoid (3), elliptical (4), round (5), cylindrical (6) as described by the Canadian Food Inspection Agency (2013). The diameter of the mature tuber was measured in millimetres (mm) with the aid of a calliper from the middle portion of the tuber. Similarly, the length of mature tubers was recorded in mm with the aid of a calliper from the proximal end to the distal end of the tuber. The width of the basal bulb was as well measured in mm with the aid of a calliper from the middle portion of the bulb. The shoot height at maturity was taken with the aid of a metre rule from soil level to the highest point of the plant in its natural orientation in centimetres (cm). The number of leaves per plant was obtained by counting the total number of true leaves on the plant (excluding bracts), including both dried and fresh ones. Leaf angle was taken as the angle between the base of the third leaf and the stalk with the aid of the protractor on a scale of 1 (0°) to 10 (90°) (Figure 2).

The colour of leaf base and leaf laminar were rated on a scale of 1 (light) to 10 (dark) green with the aid of an RGB-based colour chart; Fascicle diameter was taken in mm with the aid of a calliper as the diameter of the shoot at ground level. Culm length was measured in mm with the aid of a meter rule as the distance between the point where the culm emerges and the highest point on the culm in its natural orientation. The length of longest leaf per shoot was taken as the distance between the base of the longest leaf and its apex, with the aid of a measuring rule in mm. The width of the widest leaf per shoot was taken as the distance from one of the leaf margins end to the other, perpendicular to the mid-rib at the widest portion of the leaf. The length of the longest seed spikelet was measured as the distance between the base and the tip of the spikelet with a rule. The number of rachises per inflorescence was counted by direct observation. Length of longest rachis was measured as the distance between the base and the tip of the rachis with a rule. The number of involucral bracts per inflorescence was counted by direct observation. Length of longest involucral bract was taken as the distance between the base of the longest bract to the apex with the aid of a rule. The width of the widest involucral bract was taken as the distance from end to end of the bract margins, perpendicular to the mid-rib at the widest portion of the bract (Figure 2). Inflorescence colour was rated on a scale of 1 (light) to 10 (dark) purplish-brown.

### 2.4 Data analyses

The qualitative data collected were first summarized by simple frequencies and then analysed by the chi-square test of independence for their dependence on the four agro-ecological zones. The quantitative data collected were described with appropriate measures of central tendency and dispersion and then analysed by analysis of variance. The data were then subjected to Pearson's correlation analysis to help in scaling down the number of characters for the multivariate analysis. A total of eleven characters, including both quantitative and qualitative characters, were first standardized by dividing each data point by the maximum value for the respective characters and then used for the Principal Component Analysis which was based on the sum of squares and products. These eleven characters were further subjected to hierarchical cluster analysis with complete link methods. The qualitative data were subjected to the simple matching test while the quantitative data were subjected to the Euclidean test to form the similarity matrix before the cluster analysis. The data were analysed with GenStat Discovery Edition 4.

## 3 Results

### 3.1 Variation in qualitative characters

The skin colour of mature tubers ranged from 7 to 10 on a scale of 1 (brown) to 10 (black) (Table 2), with 8 and 9 recording the highest percentage frequencies of 40.4 % each. This gave a mean score of 8.3 ± 0.88. The colour of the leaf bases of the shoots was between 2 and 5 on the scale of 1 (light green) to 10 (green-black) with the highest percentage frequency of 57.4 % recorded by 3 and the mean score of 2.8 ± 0.82. Generally, the laminas of the leaves were greener than the bases. The lamina colour recorded were from 3 to 7 on the same scale of 1–10 used for the leaf bases. The mean score was 5.7 ± 0.89 with a score of 6 recordings the highest frequency of 46.8 %. The highest percentage frequency of 55.3 % for the inflorescence colour was recorded for score 8, followed by 7 with 25.5 %. The mean score was 7.8 ± 0.86. Leaf angle measured ranged between 4 and 8 with 6 recordings the modal percentage frequency of 42.6 %. The mean score of 6.4 ± 0.91 thus corresponded to 64^o^.

**Table 2:** Percentage frequencies of qualitative traits of purple nutsedge from southern Ghana.

Score	Skin colour of mature tubers (%)	Colour of leaf base (%)	Colour of leaf laminar (%)	Colour of inflorescence (%)	Leaf angle (%)
1	0	0	0	0	0
2	0	34	0	0	0
3	0	57.4	2.1	0	0
4	0	6.4	0	0	2.1
5	0	2.2	36.2	0	8.5
6	0	0	46.8	4.3	42.6
7	17.5	0	14.9	25.5	40.4
8	40.4	0	0	55.3	6.4
9	40.4	0	0	14.9	0
10	1.7	0	0	0	0
Mean score ± std dev.	8.3 ± 0.88	2.8 ± 0.82	5.7 ± 0.89	7.8 ± 0.86	6.4 ± 0.91

**Figure 3:**
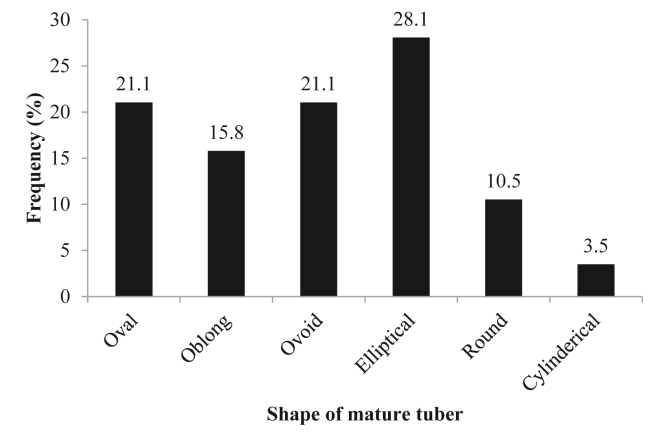
Frequency distribution of shape of mature tubers of purple nutsedge.

The mature tubers varied in their shapes with the highest proportion of 28.1 % taking the elliptical shape, followed by the ovoid and oval shapes of 21.1 % each. The least frequency percentage of 3.5 % was recorded by the tubers with the cylindrical shape (Figure 3).

#### 3.1.1 Variation of qualitative characters with agro-ecological zones

The chi-square test of independence did not show significant variation with the agro-ecological zones in respect of skin colour of mature tubers, the shape of mature tubers, the colour of leaf base, the colour of leaf laminar, the colour of inflorescence and leaf angle respectively (Table 3).

**Table 3:** Test for the dependence of qualitative characters on agro-ecological zones.

Qualitative trait	χ^2^	df	P-value
The skin colour of mature tubers	2.70	9	0.98
The shape of mature tubers	23.76	15	0.07
Colour of leaf base	3.92	9	0.98
Colour of leaf laminar	10.47	9	0.31
Colour of inflorescence	11.32	9	0.26
Leaf angle	6.83	12	0.87

#### 3.2 Descriptive statistics of quantitative characters

The descriptive statistics of the quantitative characters measured are presented in Table 4. Among the underground characteristics, the diameter of mature tubers ranged from 5.28 mm to 10.45 mm with a mean of 8.14 mm and a standard deviation of 1.22. Length of mature tuber had a mean of 13.66 mm with a standard deviation of 3.57 and ranged from 7.48 mm to 26.23 mm. The basal bulb diameter ranged from 4.29 mm to 10.41 mm with a mean of 6.38 mm and a standard deviation of 1.45. Among the vegetative shoot characters, the height of the purple nutsedge shoots ranged from 15.60 cm to 38.50 cm and had a standard deviation of 5.84, with the number of leaves ranging from 5 - 16 per plant, averaging 11.26 with a standard deviation of 2.91. The longest leaf per plant ranged from 15.50 cm to 35.70 cm with a mean of 23.87 cm and a standard deviation of 5.06. The length of the longest seed spikelet, number of rachises per inflorescence and the length of the longest rachis as 11.84 mm, 4.32 and 3.49 cm respectively with their respective standard deviations as 2.86, 0.59 and 1.05. The length of the longest seed spikelet recorded ranged from 6.00 mm to 16.00 mm whereas the number of rachises per inflorescence was within the range of 3 and 5. The maximum length of the longest rachis was 6.10 mm with 1.70 mm as the minimum.

**Table 4:** Descriptive statistics of quantitative characters measured.

Quantitative parameter	Mean	Std Error	Std Deviation	Min	Max	Confidence Level (95 %)
Diameter of mature tuber (mm)	8.14	0.16	1.22	5.28	10.45	0.32
Length of mature tuber (mm)	13.66	0.47	3.57	7.48	26.23	0.95
Diameter of basal bulb (mm)	6.38	0.21	1.45	4.29	10.41	0.42
Shoot height at maturity (mm)	26.81	0.85	5.84	15.60	38.50	1.72
Number of leaves per plant	11.26	0.42	2.91	5.00	16.00	0.85
Fascicle girth (mm)	3.01	0.10	0.65	2.06	4.33	0.19
Culm length (mm)	19.20	0.76	5.21	9.00	31.00	1.53
Length of longest leaf per shoot (mm)	23.87	0.74	5.06	15.50	35.70	1.49
Width of widest leaf per shoot (mm)	5.16	0.10	0.68	4.00	7.00	0.20
Length of longest seed spikelet (mm)	11.84	0.42	2.86	6.00	16.00	0.84
Number of Rachises per inflorescence	4.32	0.09	0.59	3.00	5.00	0.17
Length of longest rachis	3.49	0.15	1.05	1.70	6.10	0.31
Number of involucral bract per inflorescence	3.34	0.11	0.73	2.00	4.00	0.21
Length of longest involucral bract (mm)	6.52	0.31	2.11	3.50	13.50	0.62
Width widest involucral bract (mm)	3.91	0.11	0.73	3.00	6.00	0.21

#### 3.2.1 Comparison of quantitative characters of purple nutsedge from four agro-ecological zones

Tubers from the Transitional Zone recorded the least diameter of 5.43 mm and this was significantly different (p < 0.05) from the other zones (Table 5). No significant differences were observed in the diameter of mature tubers from the other zones (p > 0.05). The other underground characters, such as length of mature tubers and width of the basal bulb, did show significant differences (p > 0.05) among the agro-ecological zones even though the Transitional Zone recorded the highest length of mature tubers with 14.61 mm and the Coastal Savannah recorded the lowest length of mature tubers. For the diameter of the basal bulb, the Semi-Deciduous forest zone had the highest diameter of 7.34 mm followed by the Coastal Savannah, Tropical Rainforest and lastly the Transitional Zone in that order (Table 3).

**Table 5:** Comparison of underground characteristics of purple nutsedge from four agro-ecological zones of southern Ghana.

Agro-ecological zone	Diameter of mature tuber (mm)	Length of mature tubers (mm)	Diameter of the basal bulb (mm)
Coastal Savannah	8.37a	12.90	6.37
Tropical Rainforest	8.03a	13.64	6.32
Semi-deciduous Forest Zone	8.66a	13.06	7.34
Transitional Zone	5.43b	14.61	5.81
Standard error	0.60	2.17	0.89
%CV	14.82	26.64	22.65

Means in the same column bearing the same letter are not significantly different at P = 0.05.

In terms of vegetative above-ground characters, the Tropical Rainforest zone recorded the highest shoot height at maturity of 35.75 cm and this was significantly different (p < 0.05) from those of the Semi-deciduous Forest (24.48 cm) and transitional (24.33 cm) zones but not significantly different from the Coastal Savannah zone which recorded a mean height of 27.32 cm (Table 6). Significant differences were also observed (p < 0.05) in the number of leaves per shoot with those from Transitional Zone recording the lowest mean of 5.33 which was significantly different from all the others. Significant differences were however not observed between the other three zones. The girth of the fascicle also differed significantly among the agro-ecological zones (p < 0.05). The highest girth was recorded by collections from the Semi-deciduous Forest zone which gave a mean fascicle girth of 3.27 mm followed by the Coastal Savannah with 3.05 mm, the Tropical Rainforest 2.31 mm and finally the Transitional Zone with 2.28 mm. Significant differences were observed between the Semi-deciduous Forest zone and the Transitional Zone. A trend similar to that of the fascicle girth was observed for the length of the longest leaf: the highest was recorded by the Semi-deciduous Forest zone followed by the Coastal Savannah, Tropical Rainforest and the Transitional Zone in that order recording 25.84 cm, 23.71 cm 24.50 cm and 16.87 cm respectively. No significant difference was observed between the first three, however, they all differed significantly from the Transitional Zone (see Table 6).

**Table 6:** Comparison of shoot quantitative characteristics of purple nutsedge from the four agro-ecological zones.

Agro ecological Zone	Shoot height at maturity (cm)	Number of leaves per shoot	The girth of fascicle (mm)	Length of the longest leaf (cm)	Width of the widest leaf (mm)
Coastal Savannah	27.32ab	12.10a	3.05a	23.71a	5.07b
Tropical Rainforest	35.75a	11.67a	2.31ab	24.50a	4.00c
Semi-deciduous Forest zone	24.48b	10.58a	3.27a	25.84a	5.61a
Transitional Zone	24.33b	5.33b	2.28b	16.87b	5.33ab
Standard error	3.38	0.22	0.37	2.90	0.35
%CV	21.79	25.83	21.37	32.44	13.21

Means in the same column bearing the same letter are not significantly different at P = 0.05.

A different trend was observed for the girth of the culm where the highest girth of 28.9 cm was recorded by the Tropical Rainforest and was significantly different from those from the other zones. Differences were however not significant among the Coastal Savannah, Semi-deciduous Forest and the Transitional Zones which recorded 19.61 mm, 16.59 mm and 16.00mm respectively. The width of the widest leaf also differed significantly with the agro-ecological zones with the Semi-deciduous Forest zone recording the highest width of 5.61 mm and closely followed the Transitional Zone with 5.33 mm. While these two were not significantly different, they differed significantly from the Coastal Savannah which recorded 5.07 mm and the Tropical Rainforest which recorded 4.00 mm and were also significantly different from each other.

The number of rachises, number of involucral bracts and the length of the longest involucral bracts did not differ significantly (p > 0.05) among the agro-ecological zones (Table 7). The highest number of rachises was recorded by collections from the Semi-deciduous Forest zone with a mean of 4.67 with the Coastal Savannah recording the least with 4.21. The highest mean number of involucral bracts of 3.67 was recorded for both the Semi-Deciduous Forest and the Transitional Zones with the Tropical Rainforest recording the least of 2.67. The Transitional Zone also recorded the highest length of involucral bract of 8.17 mm whereas the least of 6.27 mm was recorded by the Semi-deciduous Forest zone. The length of spikelet from Tropical Rainforest was 15.17 mm, but this was not significantly different from those of Coastal Savannah and Transitional Zones which recorded 12.17 mm and 12.67 mm respectively. All three, however, differed significantly from the Semi-deciduous Forest which recorded a mean length of 10.00 mm. Again, the highest length of longest rachis of 4.80 mm was recorded by the Tropical Rainforest and this was significantly different from the Transitional Zone (3.83 mm), Coastal Savannah (3.49 mm) and the Semi-deciduous Forest zone (3.09 mm). The last three did not differ significantly from each other. The width of the widest involucral bract (5.33 mm) was recorded by materials from the Transitional Zone and it differed significantly from the other three zones which recorded 4.08 mm, 3.76 mm and 3.33 mm. The other three did not differ significantly from each other.

**Table 7:** Comparison of inflorescence quantitative characteristics of purple nutsedge from the four agro-ecological zones.

Agroecological zone	Length of longest spikelet (mm)	Number of rachises	Length of the longest rachis (cm)	Number of involucral bracts	Width of widest involucral bract (mm)	Length of longest involucral bract (cm)
Coastal Savannah	12.17ab	4.21	3.49b	3.24	3.76b	6.58
Tropical Rainforest	15.17a	4.00	4.80a	2.67	3.33b	5.23
Semi-deciduous Forest zone	10.00b	4.67	3.09b	3.67	4.08b	6.27
Transitional Zone	12.67ab	4.33	3.83b	3.67	5.33a	8.17
Standard error	1.59	0.11	0.61	0.08	0.37	1.28
%CV	24.12	13.75	30.09	21.87	18.63	32.44

Means in the same column bearing the same letter are not significantly different at P = 0.05.

### 3.3 Correlation between the quantitative characters measured

The highest correlation coefficient of 0.951 was observed between culm length and height of plant at maturity and this was highly significant (p < 0.01) (Table 8). Other high correlation coefficients were observed between fascicle girth and diameter of basal bulb (*r* = 0.508, p < 0.01), and length of longest spikelet and shoot height at maturity (*r* = 0.53, *p* < 0.01). The others were between length of longest rachis on one hand and shoot height at maturity (r = 0.648, p < 0.01), culm length (r = 0.610, p < 0.01) and length of longest seed spikelet (r = 0.774, p < 0.01) on the other hand. No significant correlations were found between length of mature tubers and width of widest leaf (r = 0.000, p = 0.998), diameter of mature tuber and shoot height at maturity (r = 0.008, p = 0.959) and length of longest rachis and number of involucral bracts (r = 0.002, p = 0.987) (Table 8).

**Table 8:** Correlation between quantitative characters measured on various collections of purple nutsedge.

	DMT	LMT	DBB	SHM	NLP	FAG	CUL	LLL	WWL	LLS	NRI	LLR	NIB	LLI	WWI
DMT															
LMT	.164														
DBB	.124	-.065													
SHM	.008	-.126	.086												
NLP	.449∗∗	-.170	.320∗	-.265											
FAG	.108	-.058	.508∗∗	-.163	.406∗∗										
CUL	.073	-.093	.104	.951∗∗	-.178	-.197									
LLL	.120	-.037	.061	.374∗∗	-.059	.312∗	.320∗								
WWL	-.176	.000	.094	-.279	.112	.453∗∗	-.390∗∗	.020							
LLS	-.146	-.201	.166	.530∗∗	-.071	-.171	.541∗∗	-.274	-.142						
NRI	-.097	.194	.068	.280	-.313∗	.109	.168	.202	.140	-.046					
LLR	-.157	-.194	.174	.648∗∗	-.234	-.191	.610∗∗	-.039	-.019	.774∗∗	.099				
NIB	-.112	.071	.021	-.045	-.277	.227	-.153	.114	.085	-.036	.396∗∗	-.002			
LLI	-.310∗	-.072	-.095	.475∗∗	-.432∗∗	.040	.332∗	.380∗∗	-.173	.091	.462∗∗	.196	.485∗∗		
WWI	-.492∗∗	-.034	-.043	.128	-.587∗∗	-.052	-.017	.075	.180	.017	.292∗	.213	.449∗∗	.511∗∗	

DMT: Diameter of mature tuber; LMT: Length of mature tuber; DBB: Diameter of the basal bulb; SHM: Shoot height at maturity; NLP: No. of leaves per plant; FAG: fascicle girth; CUL: Culm length; LLL: Length, longest leaf per shoot; WWL: Width, widest leaf per shoot; LLS: Length, longest seed spikelet; NRI: Rachises per inflorescence; LLR: Length, longest rachis; NIB: Number of involucral bracts; LLI: Length, longest involucral bract WWI: Width widest involucral bract.

∗∗ Correlation is significant at the 0.01 level (2-tailed).

∗ Correlation is significant at the 0.05 level (2-tailed).

### 3.4 Principal component analysis of morphological traits on purple nutsedge collection

Based on the results of the correlation analysis, the principal component analysis (Table 9) was performed on 11 out of the 21 morphological characters. The analysis grouped the characters into four latent factors (PC1, PC2, PC3 and PC4) which cumulatively accounted for a total of 82.80 % of variations observed in the samples. In the end, PC1, PC2, PC3 and PC4 account for 43.94, 20.49 10.20 and 8.17 percent respectively. The highest contribution to PC1 came from the length of the longest involucral bract with an absolute contribution of 0.821. This was followed by the number of leaves per plant with 0.242 and shoot height at maturity with 0.237. PC1 had a latent root of 7.755. Length of longest seed spikelet contributed an absolute value of 0.507 to PC2, recording the highest contribution. Other important contributors included the length of the longest rachis (0.474) the culm length, (0.429). The number of leaves per plant and the fascicle girth were the major contributors to the PC3 with 0.593 and 0.430 respectively.

**Table 9:** Principal component analysis showing contributions of morphological characters to variations in purple nutsedge collection.

Variables	PC1	PC2	PC3	PC4
Colour of leaf base	0.138	-0.047	0.096	0.104
Culm length	-0.206	-0.429	-0.277	-0.189
Length of longest involucral bract	-0.821	0.247	-0.121	-0.022
Length longest leaf per shoot	-0.138	0.092	-0.422	-0.224
Length longest rachis	-0.164	-0.474	0.122	0.286
Length longest seed spikelet	-0.109	-0.507	0.115	0.439
Number of Involucral bract per inflorescence	-0.223	0.302	0.219	0.509
Number of leaves per plant	0.242	0.012	-0.593	0.370
Shoot height at maturity	-0.237	-0.346	-0.219	-0.138
Fascicle girth	0.011	0.205	-0.430	0.450
Width widest involucral bract	-0.203	0.095	0.247	0.105
Latent roots (Eigen Values)	7.755	3.617	1.801	1.442
Percentage Variation	43.94	20.49	10.20	8.17
Cumulative % Variation	43.94	64.43	74.63	82.80

### 3.5 Cluster analysis of collections

**Figure 4:**
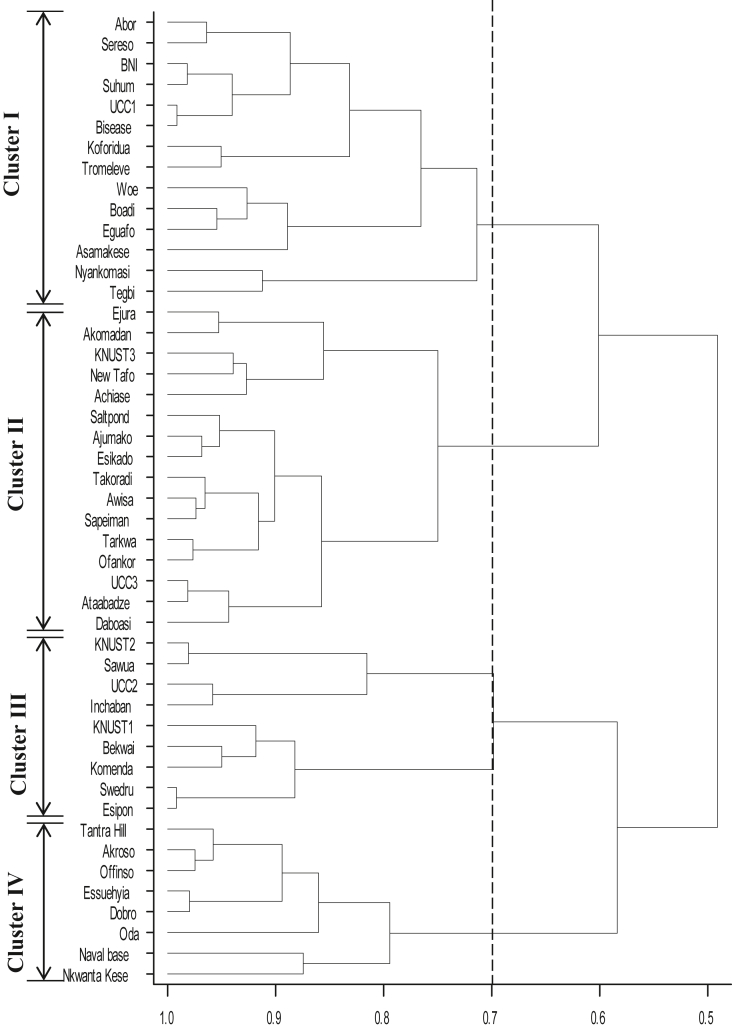
Dendrogram of the purple nutsedge collection based on morphological characters cut at 0.7 similarity coefficient.

The similarity coefficients for the cluster analysis ranged from 0.5 (dissimilar) to 1.0 (similar). At 0.5 similarity coefficient, all the 48 collections were put into one cluster, however, at 0.7, they further divided into four clusters. The four clusters each comprised collections from the various agro-ecological zones suggesting the clustering did not depend on agro-ecological conditions (Figure 4).

## 4 Discussion

### 4.1 Comparison of morphological characteristics with those in other countries

The characteristics of underground structures of the purple nutsedge were consistent with those reported from other locations across the globe (Wills, 1998; Riemens et al., 2008). The skin colour of the tubers ranged from brown to black and was of diverse shapes: oval, oblong, ovoid, elliptical, round and cylindrical. Ovoid was the modal shape. Both skin colour of tuber and shape of tubers did not show a significant dependence on the source agro-ecological zones. These observed tuber characteristics confirm the assertion by Sholedice and Renz (2006) that tubers of the purple nutsedge are round to oblong and often irregular in shape and are covered with red to brown scales.

The samples had pale green to spring green leaf bases with forest green leaf laminar. The inflorescence was generally dark purple to reddish-brown with an average leaf angle of 64^˚^. Again, these characteristics did show a significant dependence on their respective agro-ecological zones and hence indicates that variations were random or may have been caused by some other factors. The described characteristics conform to earlier reports (Riemens et al., 2008; Stoller and Sweet, 1987; Wills, 1987; Bryson and DeFelice, 2009). It is generally known that green colouration of leaves and leaf bases are indicative of the presence of chlorophyll pigments which is important for photosynthesis in plants (Terashima et al., 2009).

Despite the conformity of the qualitative characteristics to those from other countries, most of the quantitative characteristics differed substantially. The results showed that the shoot height of the weed in the study area was much lower than those from other geographical locations as reported by Wills (1998) except Western Samoa, Tanzania, Sudan, Malaysia, Japan and Greece, which recorded figures within the range of heights for the study area. Similarly, substantially lower results were obtained for culm length, the width of widest leaf, number of rachises per inflorescence, length of the longest rachis, number of involucral bracts per inflorescence, length of involucral bracts and width of widest involucral bracts. The width of the widest leaves, nonetheless, was within the reported range and this also agreed with the report by Bryson and DeFelice (2009) who gave a range of 0.5mm–12.7 mm. In essence, the purple nutsedge samples from southern Ghana were generally smaller in size than those reported from other geographical location across the globe. This could point to an ecotypic adaptation by the weed to the conditions in Ghana.

### 4.2 Variation of characters with agro-ecological zones

There was no significant relationship between qualitative characters and the four agro-ecological zones, indicating low qualitative diversity in the weed within Southern Ghana. The geographic origin of plants could influence crops’ morphology if the geographic locations have different climatic conditions and soil (Adu et al., 2019; Narayanan et al., 2014). Given that all the plant samples used in the present study originated from Southern Ghana with, to a large extent, comparable climatic conditions, it unsurprising that the materials did not show a wide diversity in the qualitative traits.

Even so, significant differences were observed in the quantitative traits. Purple nutsedge collections from transitional zone consistently recorded significantly lower value for quantitative traits, both above-ground and below-ground. Considering that, the quantitative traits mostly are indicators of growth, the observation suggests that the samples from the transitional zone gave poorer growth compared with the others. Several reports have shown that purple nutsedge collections from wet lowlands have significantly higher biomass than those from drier uplands (Baltazar et al., 1997; Pena-Frontera et al., 2009; Fuentes et al., 2010). Therefore, it was not surprising to observe that collections from the two forest zones which have relatively higher amounts of rainfall recorded higher biomass than those obtained from the transitional zone which is comparatively drier. Crop production in the coastal savanna mainly focuses on the cultivation of vegetables and employs irrigation, thus keeping the soils wet throughout the year in contrast with the transitional zone that focuses on field crops with little or no irrigation. Therefore, the relatively low growth recorded by the samples from the transitional zone could be a result of the drier soils that characterize farming in that area.

Qualitative and quantitative traits may be independently associated with changes in climate factors such that small changes in climatic factors could result in changes in quantitative traits but not in qualitative traits of the weed. Thus, small variations in some climatic factors between the agro-ecological zones have resulted in varying morphological and/or physiological adaptions of the weeds. This is confirmed by the fact that the qualitative characteristics of purple nutsedge in the study area neither varied with those from other countries nor among the agro-ecological zones. Adaptations to different agro-ecological conditions in purple nutsedge have been reported in several locations (Okoli et al., 1997; Casimero et al., 1999; Chavez and Moody, 1986; Komai et al., 1983; Jha and Sen, 1980). These adaptions serve as survival mechanisms and promote growth and proliferation of the weed under the respective conditions, particularly rainfall pattern and distribution, and soil characteristics. They also enhance their competitive ability and give them the ability to compete well with crops on vegetable fields.

### 4.3 Factor and cluster analysis of purple nutsedge collection

The Principal Component analysis gave four latent factors as the major components determining variations in the collections. The traits which resolved and contributed heavily to variation of PC1, including length of the longest involucral bract, number of leaves per shoot, number of involucral bracts, shoot height at maturity and the culm length are all photosynthetic structures of the plant. Thus, the first and most important component contributing to variation in purple nutsedge in southern Ghana may comprise the photosynthetic structures. This is unsurprising given the plant's C_4_ photosynthetic cycle which makes it require and utilize high radiation and temperature efficiently (Lima et al., 2015). Thus, morphological variations are likely to occur with variations in the amount of radiation and temperature as an adaptation to the immediate environment. The second dimension explains mainly primary growth-related traits including plant height, while the third dimension appears to be related to secondary growth and leaf-related traits such as fascicle girth and leaf numbers. These traits are more likely to be useful in differentiating collections of the weed and therefore warrant consideration in efforts to design management strategies for the weed.

Four major groups of the plant were obtained from the cluster analysis. These four clusters did not show any trend concerning the locational sources of the samples nor their original agro-ecological zones. A close look at the collections from the University of Cape Coast research stations (UCC1, UCC2 and UCC3) showed that the three did not occur in the same cluster; they occurred in clusters I, III and II respectively despite the similarities in the environmental conditions in their locations from which they were obtained. Again, whereas two of the collections from the Kwame Nkrumah University of Science and Technology, KNUST1 and KNUST2, occurred in cluster III, KNUST3 occurred in cluster II despite the environmental similarities of their sources. These suggest that there is a large variation in the collections from the same agro-ecological zone, and seem to support the suggestion that variations are more likely to occur in response to radiation and temperature differences in the immediate environment.

In summary, despite the differences observed in some of the quantitative traits, all the qualitative traits did not vary with the samples collected from the four agro-ecological zones. Further, clustering was unrelated to the agro-ecological zones. This suggests that minimal genetic diversity existed among the samples, thus little to no amount of speciation has occurred in the weed in southern Ghana. However, some level of ecological adaptation seems to have occurred in the study area.

### 4.4 Implications for weed management

The similarities and differences observed can influence the effectiveness of weed management approaches employed. Bangarwa et al. (2008) observed that large and medium-sized tubers remained stable under season-long management systems but small-sized tubers were likely to deplete over time. Bielinski, Morales-Payan, Stall and Bewick (1997) also found that for smaller tubers, average shoot number and dry weight of regrowth followed a decreasing pattern over time. This pattern is seen in the characteristics of tubers from the transitional zone which gave smaller tubers as well as shorter shoots. Accordingly, one would expect that the purple nutsedge in the transitional zone may become less noxious with time and later become depleted if regular and consistent management of the weed is carried out since the tubers are smaller in size. This observation may be responsible for the less noxiousness of the weed in some part of the study area that was reported by Tachie-Menson (2016). Islam et al. (2005) suggested that strategies that reduce tuber survival are less costly than those that aim at reducing tuber production and this can be explored to effectively control the weed in the transitional zone at lower costs.

Again, larger leaf surfaces and higher total plant biomass as recorded by the three agro-ecological zones could aid in the intercept of more herbicides than narrow and smaller shoots. This could make the purple nutsedge in such places more prone to herbicides than those in the transitional zone, which were generally smaller in size.

## 5 Conclusion

The qualitative traits of the purple nutsedge samples from Ghana were similar to those reported from other countries. These traits also did not vary with the four agro-ecological zones in the study area. Significant variations, however, were observed between agro-ecological zones for some quantitative variables, which were also indicators of growth. The variable that showed significant differences include the diameter of tubers, shoot height at maturity, length of mature tubers, the width of the basal bulb, number of leaves per shoot, longest rachis and width of the widest involucral bract. The samples were also considerably smaller when compared to those reported from the other countries concerning several quantitative traits. This variation suggests some form of morphological adaptation of the purple nutsedge to the conditions in Ghana and further in the agro-ecological zones. Samples from the transitional zone gave significantly lower values for most of the growth variables and this could lead to the weed becoming less noxious in that agro-ecological zone with time. The principal components that determined variations in purple nutsedge collections were photosynthetic structures (involucral bracts and leaves), plant height and leaf characteristics. The collections clustered into four major classes at 0.7 similarity coefficient but these clusters were neither related to their geographical sources nor their agro-ecological zones.

## Declarations

### Author contribution statement

J. W. Tachie-Menson; J. N. Buah: Conceived and designed the experiments; Performed the experiments; Analyzed and interpreted the data; Contributed reagents, materials, analysis tools or data; Wrote the paper.

M. O. Adu; E. Afutu: Analyzed and interpreted the data; Contributed reagents, materials, analysis tools or data; Wrote the paper.

### Funding statement

This research did not receive any specific grant from funding agencies in the public, commercial, or not-for-profit sectors.

### Data availability statement

Data will be made available on request.

### Declaration of interests statement

The authors declare no conflict of interest.

### Additional information

No additional information is available for this paper.
